# Morphometric Analysis of Permanent Canines: Preliminary Findings on Odontometric Sex Dimorphism

**DOI:** 10.3390/ijerph19042109

**Published:** 2022-02-13

**Authors:** Abdulelah A. Alanazi, Abdulmohsen Musaad Almutair, Abdullah Alhubayshi, Abdullah Almalki, Zuber Ahamed Naqvi, Abdullah Alassaf, Basim Almulhim, Sara Ayid Alghamdi, Sreekanth Kumar Mallineni

**Affiliations:** 1 College of Dentistry, Majmaah University, Al-Majmaah 11952, Saudi Arabia; dr.abdulelah.as@gmail.com (A.A.A.); dr.abdulmohsen99@gmail.com (A.M.A.); abd.allah2012@hotmail.com (A.A.); 2 Department of Preventive Science, College of Dentistry, Majmaah University, Almajmaah 11952, Saudi Arabia; ae.almalki@mu.edu.sa (A.A.); zuber2881@gmail.com (Z.A.N.); am.assaf@mu.edu.sa (A.A.); b.almulhim@mu.edu.sa (B.A.); sa.mohammed@mu.edu.sa (S.A.A.); 3 Center for Transdisciplinary Research (CFTR), Saveetha Dental College, Saveetha Institute of Medical and Technical Sciences, Saveetha University, Chennai 600077, India

**Keywords:** sex dimorphism, inter-canine width, standard canine index, canine, dimorphism

## Abstract

Aim: This study aims to investigate the morphometrics of permanent canines in establishing sexual dimorphism in the native Arabian population. Methods: Thirty (male = 12; female = 18) native Arabian subjects, with ages ranging between 20–45 years. The mesiodistal (MD), cericoincisal (CI) and labiolingual (LL) widths of the teeth 13, 23, 33, and 43 and the inter-canine distance in maxillary (MaxICW) and mandibular (ManICW) arches were measured using a digital caliper. The gran method was used for establishing sex dimorphism among the study subjects. Descriptive statistics were employed using SPSS version 20.0 (Armonk, NY, USA, IBM Corp.). Results: The comparison of either of the measurements (MD, CI, LL, MaxICW and ManICW) were shown to be statistically significant (*p* > 0.05). The overall mean values of teeth 13, 23, 33, and 43 for CI, LL, MaxICW, and ManICW were lower for females than males (*p* > 0.05). The MD width was higher in females than that of males (*p* > 0.05). The sex dimorphism value for teeth 13, 23, 33, and 43 were 0.98, 0.99, 1, and 0.99, respectively. The standard canine index was high for mandibular teeth and lower for mandibular teeth, and SCI values for teeth 13, 23, 33, and 43 were 0.219, 0.218, 0.257 and 0.256, respectively. Conclusion: The morphometrics of permanent canines are helpful in sex determination with the aid of odontometric analysis.

## 1. Introduction 

Teeth are the hardest mineralized tissue in the human body and are more resistant to post-mortem obliteration than tissues of other parts of the human body. This oddity of teeth is helpful to detect the sex of fatalities during mass disasters [1,2,3,4,5]. Sex identification plays a vital role in any investigation performed in forensics [6,7,8,9,10,11]. This could be possible by DNA analysis [12], osteometry [13] and odontometric analysis [14]. Among these methods, DNA analysis has been reported to have an overall higher level of accurate results. Nevertheless, in many situations DNA analysis may not be possible due to its cost effectiveness, DNA extraction techniques, and moreover it necessitates highly qualified personnel [12,15,16]. During mass disasters, the identification of the sex of a dead individual is an imperative process in forensics [2,6]. The published literature shows that the teeth are established as an essential material in sex determination, maybe due to their resistance to chemical and mechanical agents in the post-mortal process [8,10]. The difference in size, appearance and stature is called dimorphism [3]. The variations in tooth shape and size between the sexes are also called sexual dimorphism.

Numerous approaches had have been reported to use sex determination in forensic and anthropological investigations. Incisors, molars, canines, and mandibular parameters have been extensively used in sex dimorphism studies [8,9,10,11,12,13]. Canines are extensively used for sex identification because of their durability in the oral cavity and morphological variance [16]. The majority of researchers use the mandibular canine index that is considered as a reliable source for sex determination. Mesiodistal and labiolingual widths, inter canine width (ICW) and the canine index of the permanent teeth are the most frequently used in determining sex [17,18,19,20,21,22]. Among them, the majority of researchers prefer mandibular canines for the assessment. None of the studies used both maxillary and mandibular canines for sex determination in the Arabian population. Thus, there is a lacuna regarding the sex determination in the native Arabian population using permanent canines. Therefore, there is a need to determine odontometric standards for related sex of the individual in the Arabian population. Nevertheless, the study aims to analyze sex dimorphism in the mesiodistal (MD), cervicoincisal (CI), labiolingual (LL), and ICW of the permanent maxillary and mandibular canines.

## 2. Material and Method

### 2.1. Sample Collection and Setting

The material of the present study consists of 30 maxillary and mandibular diagnostic dental casts of 30 subjects (12 males and 18 females) of ages ranging from 20 to 45 years. Dental casts belonged to native Arabian subjects; presence of both maxillary and mandibular canines, lack of canines’ developmental shape anomalies, and without crowding, dental/occlusal abnormalities in canines were included in the study. Subjects with physiological or pathological wearing of teeth, malaligned teeth, crowding, rotation or malocclusion, any history of restoration, orthodontic treatment or trauma, spacing and partially erupted teeth and absence of any permanent canine were excluded from the study sample. The MD (maximum expanse between the proximal aspects of the crown), CI (from tip of the crown to cementoenamel junction) and LL (labiolingual or buccolingual measured with the caliper held at right angles to the MD width) widths of maxillary and mandibular canines, and the ICW in both arches were measured by a single examiner using a digital caliper (0–150 millimeter (mm)/0–6”) (INSIZE Company, Jiangsu, China). The ICW is described as the linear distance between cusps of right and left canines in maxillary (MaxICW) and mandibular arch (ManICW). The same dentist carried out all measurements to eliminate inter-observer error.

### 2.2. Ethical Clearance and Informed Consent

The ethical approval (MUREC-APR-21/Com-2021/33-2) was obtained from the Deanship of scientific research, Majmaah University, Al-Majmaah, Saudi Arabia and informed consent was obtained from all the participants.

### 2.3. Statistical Analysis

The attained dimensions analysis was performed using the SPSS (version 20.0 Armonk, NY, U.S.A., IBM Corp). An independent Student’s *t*-test was used to define the mean difference in the MD, CI, and LL widths of canine and ICW dimensions among males and females. The descriptive charts for the mean and medians of the canine index were also evaluated. The normality test for the data was conducted with the Shapiro–Wilk test, in which the values were found to be equally distributed. The confidence interval was set to 95%, and the acceptable error border was set to 5%. The mean and standard deviation of CI (canine index) were derived separately for males and females, and a cutoff point to distinguish the sex of the individual, termed “Standard CI,” was calculated as follows:

Standard CI = ([mean male CI − SD] + [mean female CI + SD])/2.

A CI value less than or equal to the standard CI means the subject is considered female. The subject is deemed male if the CI value was more than the standard CI. Sex dimorphism in the right and left mandibular canines was calculated using the formula given by Garn et al. (1967) [23,24] as follows:

Sexual dimorphism = (*X*m ÷ *X*f − 1) × 100 (Mean male canine width (*X*m) and mean female canine width (*X*f)). The obtained dimensions were subjected to statistical analysis to assess sex differences using an unpaired *t*-test. Statistical analysis was performed regarding MD, CI, LL, MaxICW and Man ICW, and canine index for teeth 13, 23, 33, and 43, and standard CI and sexual dimorphism were also calculated. Percentage accuracy of reporting sex identity by this method was then checked as the actual sex of each subject was known by comparing means and median for teeth 13, 23, 33, and 43.

## 3. Results

### 3.1. Descriptive Statistics

Descriptive statistics showing mean and the standard deviation for the different parameters of male and female in maxillary and mandibular canines in Table 1. Males were observed with comparatively lesser mean values of mesiodistal width for teeth 13, 23, 33, and 43 than the female subjects. In the case of cervicoincisal width, males showed higher mean values for teeth 13, 23, 33, and 43 compared to females, while in the case of labiolingual width, both males and females had almost similar mean values. The mean intra canine width in the maxillary arch was 34.87 mm in males and 34.55 mm in females, while mandibular intra canine width for males was 26.39 mm and 26.38 mm for females. The equal variance assumed for MD width (Table 2), CI width (Table 3) and LL width (Table 4) of teeth 13, 23, 33, and 43, measured and the result was not statistically significant. The equality of mean difference for ICW for maxillary arch and mandibular arch was measured, and the findings were not statistically significant (Table 5).

### 3.2. Sex Dimorphism

Sex dimorphism values for teeth 13, 23, 33, and 43 were 0.98, 0.99, 1 and 0.99, respectively (Table 6). The mean difference of the canine index was carried out for teeth 13, 23, 33 and 43 and none of the differences were found to be statistically significant (Table 7). Table 7 presents the difference between the two mean values for males and females regarding both arches, and the result was not statistically significant. The canine index was higher for tooth 33 and lower for tooth 13. The mean value of the right maxillary canine index in males and females was 0.218 and 0.223, respectively. The left maxillary canine index in males and females was 0.217 and 0.222, respectively.

### 3.3. Percentage Accuracy and Standard Canine Index (SCI)

**Figure 1:**
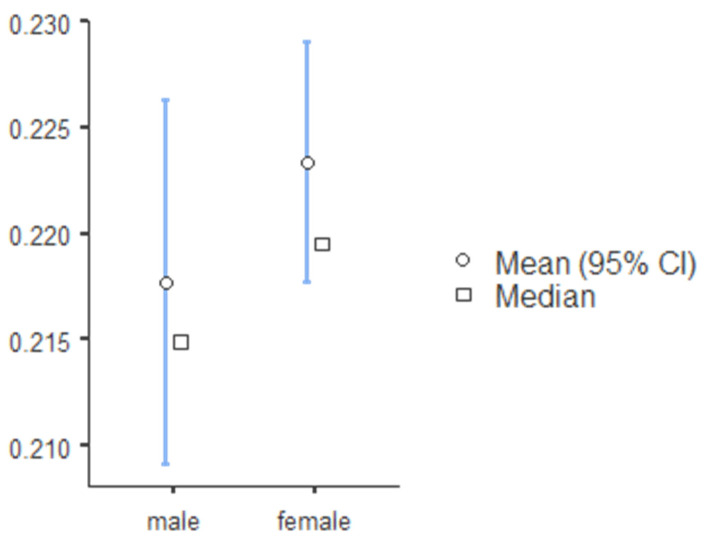
The descriptive chart of mean and median of permanent maxillary right canine index.

**Figure 2:**
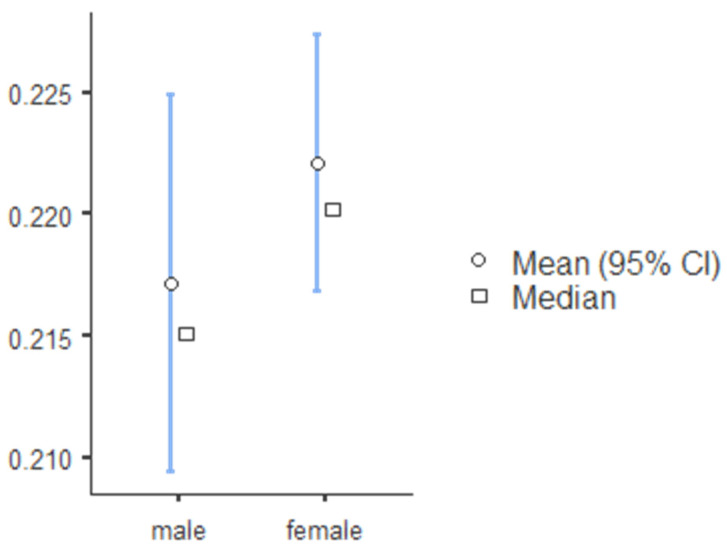
The descriptive chart of mean and median of permanent maxillary left canine index.

**Figure 3:**
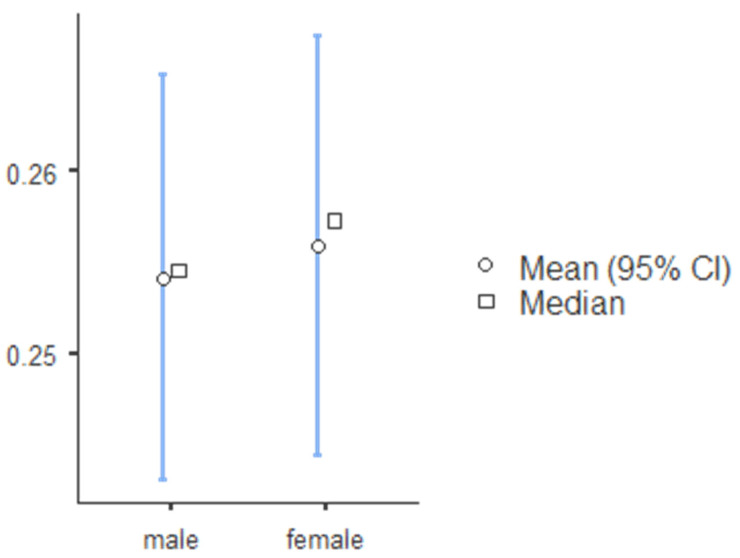
The descriptive chart of mean and median of permanent mandibular right canine index.

**Figure 4:**
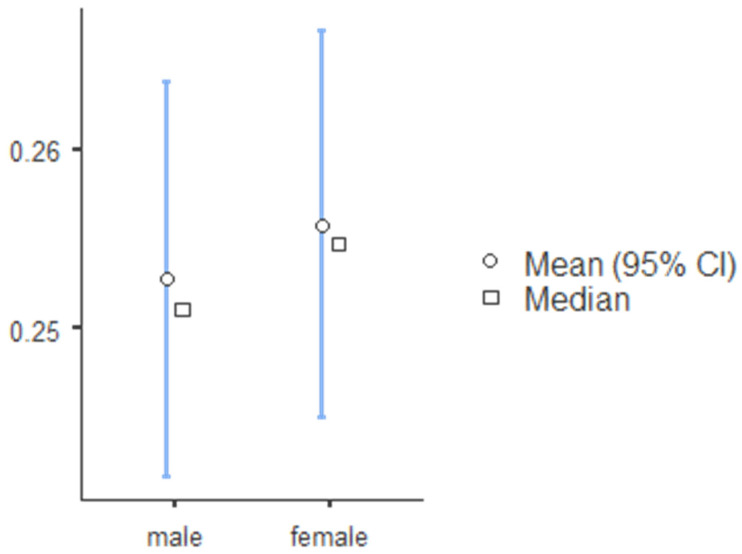
The descriptive chart of mean and median of permanent mandibular left canine index comparing sex.

The standard canine index was higher for mandibular teeth and lower for mandibular teeth, and SCI values for teeth 13, 23, 33, and 43 were 0.219, 0.218, 0.257 and 0.256, respectively (Table 8). The percentage accuracy of reporting sex identity was evaluated, and the mean and median canine index values for males and females in the maxillary and mandibular arch are illustrated in Figure 1 (tooth 13), Figure 2 (tooth 23), Figure 3 (tooth 33), and Figure 4 (tooth 43). The standard CI of the maxillary and the mandibular arch is presented in Table 8. Subjects with more significant CI values than SCI were males, and subjects with lower CI values than SCI were females.

## 4. Discussion

Morphological traits and anthropometric techniques are valuable resources in forensic sciences. Sex identification has become one of the essential parameters in any forensic investigation. Teeth have been considered by the majority of the researchers [11,12,13,14,15,16,17,18,19,20,21,22] for sex determination with the aid of odontometric analysis. The odontometric parameters offer an alternative, easy and dependable approach to sex determination [25,26,27,28,29,30,31]. Prior investigations have confirmed that permanent teeth could provide an ideal sample for dental measurements [32,33,34]. Accordingly, the present study evaluated the sexual dimorphism of four odontometric parameters (MD, LL, CI, and ICW) in the Arabian population. In the present study, reverse sex dimorphism was found as mesiodistal diameter was higher in females than males without such an extensive distinction, consistent with prior studies [35,36]. Previous studies [13,37,38,39,40] reported that the males showed higher mean values for MD diameter for each canine compared to females in both maxillary and mandibular arch. The present study showed that the mean value of CI in males is higher than in females, and the result was consistent with earlier published studies [13,35,40]. An Indian study [35] observed that the canines have been the most dimorphic tooth and have been notably more prominent in the males in MD and LL dimensions. Similarly, the LL dimensions in the present study were higher in the males than in the females, and the comparison did not show any statistically significant difference. The ICW values of males were higher than females for each maxillary and mandibular canine. These findings are in agreement with a prior survey conducted on a Saudi Arabian population [41]. Alrifaiy et al. [41] reported that the ICW values for the maxillary arch and mandibular were higher in the males than the females, and the findings were statistically significant (*p* < 0.00001). However, in the present study, the findings were not statistically significant. Based on the results from the present study, the right maxillary canine width displays the most sexual dimorphism (0.98); the findings were not statistically significant. Similarly, a study [42] with 720 pre-treatment orthodontic casts in a Saudi Arabian population aged 13–20 years observed no statistically significant difference between the left and right canines. The authors also suggested that the tooth’s dimension on one side could be genuinely representative when the contralateral measurements were unobtainable [40]. In the present study, the difference was evident among the right and left sides of the canine index that was measured.

Various researchers performed on different teeth to establish dental sex dimorphism. The majority of the studies used canines [22,40,41,43] for sex dimorphism, and some of the researchers also used first permanent molars [31,44,45], incisors [46], and all teeth [47] for the analysis. In the present study, the authors used morphometrics of canines to study sex dimorphism. The literature search showed that a few researchers [13,37,39] studied maxillary canine measurements for sex dimorphism, and a few studies [25,47] utilized mandibular canine analysis. At the same time, both maxillary and mandibular canine measurements were also used in a couple of studies [19,41]. In the present study, the authors used both maxillary and mandibular canine measurements for the analysis. The mesiodistal and cervicoincisal widths and inter canine widths were used to analyze sex dimorphism in previously published studies [13,19,25,37,39,46]. Nevertheless, a Serbian study [38] used labiolingual measurements to study sex-based odontometrics of the permanent canines. In the present study, the authors used all three measurements (mesiodistal, cervicoincisal, and labiolingual widths) and intra canine width for the analysis. To the best of our knowledge, this is the first study of its kind to use all three measurements. A Nepalese study [48] used digital jaw radiography in their odontometric analysis. A recent Indian study [49] used suture analysis for their odontometric analysis. The canine morphometrics involving MD, CI, LL, and ICW were used to identify sex dimorphism in the present study. To the best of the authors’ knowledge, this is the only study that used these three measurements for the analysis, and their measurements found varied in males and females. A Serbian study (39) used Moorrees and Reed’s method [50] to determine measurements of the canines in the present study; the authors used the Gran [22,23] method.

The majority of the studies reported on sex dimorphism used dental casts for the morphometric analysis [13,19,22,23,25,37,39,41,42,46]. The morphometric analysis of teeth was performed using either casts or intraoral measurements. Nonetheless, Barrett et al. [51] reported that intra-oral measurements are less accurate for morphometric analysis. Subsequently, Kaushal et al. [52] reported no significant difference among the measurements calculated from dental casts and intra-orally. The findings are inconsistent with an Indian study [37], which reported sex determination using the mandibular canine index and standard canine index. Nonetheless, the maxillary canine index confirmed poor sex predictability. Sex dimorphism value for teeth 13, 23, 33, and 43 were 0.98, 0.99, 1 and 0.99, respectively. This result reports that tooth 33 had more dimorphism among the males and females than teeth 13, 23, and 43. The standard canine index was high for mandibular teeth and lower for mandibular teeth, and SCI values for teeth 13, 23, 33, and 43 were 0.219, 0.218, 0.257, and 0.256, respectively. This explains the comparatively higher canine index for tooth 33 in native Arabians. The authors used dental casts for the morphometric analysis in the present study, and only linear measurements were utilized. Such studies are recommended due to their simplicity, reliability, and inexpensiveness. A smaller sample size and a single examiner involved in measurements would have led to some bias within the dimensions and results. Henceforth, the generalization of the study’s findings is not possible. The tooth dimensions of every population differ, influenced by racial, environmental, and cultural factors. Furthermore, this is the first study that used morphometric analysis involving MD, CI, LL, and IC widths, and CI to the best of the authors’ knowledge. These findings can be used as a reference manual for further canine metrics studies.

## 5. Conclusions

The study suggests the linear measurements of permanent maxillary and mandibular canines as an additional method to study sex dimorphism in forensics. The study also established the presence of sexual dimorphism among males and females in the native Arabian population. Further studies are needed, with a large sample size to establish sex dimorphism in the Arab population based on the canine metrics.

## Figures and Tables

**Table 1:** The odontometrics of permanent canines based on sex.

Parameter	Sex	N	Mean (mm)	Std. Deviation (mm)	Std. Error Mean (mm)
MD13	Male	12	7.579	0.531	0.153
	Female	18	7.700	0.460	0.108
MD23	Male	12	7.562	0.521	0.150
	Female	18	7.655	0.406	0.095
MD33	Male	12	6.675	0.475	0.137
	Female	18	6.705	0.328	0.077
MD43	Male	12	6.637	0.471	0.136
	Female	18	6.705	0.311	0.073
CI13	Male	12	9.837	0.808	0.233
	Female	18	9.200	1.356	0.319
CI23	Male	12	9.854	0.861	0.248
	Female	18	9.472	1.191	0.28
CI33	Male	12	9.987	0.922	0.266
	Female	18	9.466	1.120	0.264
CI43	Male	12	10.012	1.161	0.335
	Female	18	8.777	2.308	0.544
LL13	Male	12	2.904	0.355	0.102
	Female	18	2.933	0.469	0.110
LL23	Male	12	2.970	0.31	0.089
	Female	18	2.966	0.489	0.115
LL33	Male	12	2.733	0.339	0.098
	Female	18	2.667	0.515	0.121
LL43	Male	12	2.720	0.348	0.101
	Female	18	2.711	0.534	0.126
MaxICW	Male	12	34.879	2.299	0.663
	Female	18	34.511	1.775	0.418
ManICW	Male	12	26.391	2.627	0.758
	Female	18	26.388	2.311	0.545

MD = Mesiodistal width; CI = Cervicoincisal width; LL = Labiolingual width; Max = Maxillary arch; Man = Mandibular arch; ICW = Intracanine width; Tooth numbering in FDI system.

**Table 2:** The mean difference between two mean using *independent sample t-test* or the mesiodistal width of maxillary and mandibular canine.

Measurement	Variance	*t*	Mean Difference	Std. Error Difference	95% Confidence Interval of the Difference		*p*-Value
					Lower	Upper	
MD13	Equal variances assumed	−0.662	−0.12	0.182	−0.495	0.253	0.51 ^NS^
MD23	Equal variances assumed	−0.549	−0.093	0.169	−0.44	0.254	0.59 ^NS^
MD33	Equal variances assumed	−0.209	−0.03	0.146	−0.33	0.269	0.84 ^NS^
MD43	Equal variances assumed	−0.478	−0.068	0.142	−0.36	0.224	0.64 ^NS^

MD = Mesiodistal width; NS = non-significant (*p* > 0.05); Tooth numbering in FDI system.

**Table 3:** The mean difference between two mean using *independent sample-t-test* for the cervicoincisal of maxillary and mandibular canine.

Measurement	Variance	*t*	Mean Difference	Std. Error Difference	95% Confidence Interval of the Difference		*p*-Value
					Lower	Upper	
CI13	Equal variances assumed	1.459	0.63750	0.437	−0.257	1.532	0.16 ^NS^
CI23	Equal variances assumed	0.955	0.38194	0.4	−0.438	1.201	0.35 ^NS^
CI33	Equal variances assumed	1.334	0.52083	0.390	−0.278	1.320	0.19 ^NS^
CI43	Equal variances assumed	1.707	1.23472	0.723	−0.246	2.716	0.09 ^NS^

CI = Cervicoincisal width; NS = non-significant (*p* > 0.05); Tooth numbering in FDI system.

**Table 4:** The mean difference between two mean using *independent sample-t-test* for the labiolingual (LL) width of maxillary and mandibular canine.

Measurement	Variance	*t*	Mean Difference	Std. Error Difference	95% Confidence Interval of the Difference		*p*-Value
					Lower	Upper	
LL13	Equal variances assumed	−0.183	−0.029	0.1594	−0.356	0.297	0.85 ^NS^
LL23	Equal variances assumed	0.026	0.004	0.1597	−0.323	0.331	0.97 ^NS^
LL33	Equal variances assumed	0.393	0.066	0.1694	−0.28	0.413	0.69 ^NS^
LL43	Equal variances assumed	0.055	0.009	0.1753	−0.349	0.369	0.95 ^NS^

LL = labiolingual/buccopaltal width; NS= Non-Significant (*p* > 0.05); Tooth numbering in FDI system.

**Table 5:** The mean difference between two mean using *independent sample-t-test* for the maxillary and mandibular inter-canine width (ICW).

ICW	Variance	*t*	Mean Difference	Std. Error Difference	95% Confidence Interval of the Difference		*p*-Value
					Lower	Upper	
Max ICW	Equal variances assumed	0.494	0.368	0.745	−1.15	1.89	0.62 ^NS^
Man ICW	Equal variances assumed	0.003	0.003	0.909	−1.86	1.87	0.1 ^NS^

Max = maxillary; Man = mandibular; ICW = Inter-canine width; NS = Non significant.

**Table 6:** Sex dimorphism value of permanent canines.

Tooth	Sex Dimorphism	Value
13	0.98	−0.02
23	0.99	−0.01
33	1.00	0.00
43	0.99	−0.01

Tooth numbering in FDI system.

**Table 7:** The mean difference between two mean using *independent sample-t-test*.

Tooth	*T*	Mean Difference	Std. Error Difference	95% Confidence Interval		*p*-Value
				Lower	Upper	
CIMax13	−1.121	−0.005	0.005	−0.016	0.004	0.27 ^NS^
CIMax23	−1.072	−0.004	0.004	−0.014	0.004	0.29 ^NS^
CIMan33	−0.205	−0.001	0.008	−0.019	0.015	0.83 ^NS^
CIMan43	−0.374	−0.003	0.008	−0.019	0.013	0.71 ^NS^

Max = maxillary; Man = mandibular; CI = Canine Index; ^NS^ = non-significant (*p* > 0.05) tooth numbers are stated in the FDI system.

**Table 8:** Standard canine index of permanent canines in maxillary and mandibular arch.

Tooth	SCI
CIMax13	0.219
CIMax23	0.218
CIMan33	0.257
CIMan43	0.256

Max = maxillary; Man = mandibular; SCI = Standard Canine Index; tooth numbers are stated in the FDI system.

## Data Availability

The data that support the findings of this study are available from the corresponding author upon reasonable request.
